# Definition, analysis, reporting, and interpretation of perioperative bleeding in randomized controlled trials: a methodological systematic review

**DOI:** 10.1186/s13741-026-00715-z

**Published:** 2026-07-02

**Authors:** Yasaman Mohammadi Kamalabadi, Guangyong Zou, Maha El-Shimy, Natalie Mariam Zitoun, Justyna Bartoszko, Pavel S. Roshanov

**Affiliations:** 1 Department of Epidemiology and Biostatistics, Schulich School of Medicine and Dentistry, Western University, London, Canada; 2 Schulich School of Medicine and Dentistry, Robarts Research Institute, Western University, London, Canada; 3 Alimentiv Inc., London, Canada; 4 London Health Sciences Centre, Victoria Hospital, London, Canada; 5 London Health Sciences Centre Research Institute, London, Canada; 6 Department of Anesthesia and Pain Management, Sinai Health System, Women’s College Hospital, University Health Network, Toronto, ON Canada; 7 Division of Nephrology, Department of Medicine, Western University, London, Canada; 8 Population Health Research Institute, Hamilton, Canada

**Keywords:** Perioperative bleeding, Blood loss measures, Surgical complications, Surgical bleeding, Outcome definition, Outcome reporting, Outcome assessment

## Abstract

**Background:**

Perioperative bleeding is a common surgical complication and a frequently used outcome in randomized trials; however, its definition and analysis are not straightforward and have important implications for trial design, statistical power and interpretation of treatment effect. This review aimed to summarize how perioperative bleeding was defined in randomized controlled trials, along with sample size considerations, analytic approaches, and interpretation practices.

**Methods:**

On April 9th, 2025, we systematically searched the CENTRAL database for randomized trials in surgical settings published after January 2024 that evaluated perioperative bleeding as the primary outcome. Two reviewers independently screened and extracted data. Where appropriate, we categorized findings into conceptually similar domains and applied descriptive analysis.

**Results:**

We included 115 trials reporting 116 primary bleeding outcomes and identified 36 unique definitions, with assessment periods ranging from intraoperative to more than 30 days postoperatively. Volumetric or gravimetric measures were most common (*n* = 46 [39.6%]), whereas patient-reported events (*n* = 5 [4.3%]) and clinical management of blood loss (*n* = 4 [3.4%]) were rare. Most trials reported a sample size calculation based on the primary outcome (*n* = 90 [78.3%]), but over one-third lacked effect-size justification (*n* = 34 [37.8%]). Analyses were predominantly parametric (*n* = 69 [59.5%]), while a few adjusted for baseline covariates (*n* = 18 [15.6%]). Interpretation in superiority trials relied mainly on statistical significance (*n* = 49 [47.6%]). Among noninferiority trials, noninferiority was often claimed without prespecified margins (*n* = 4 [33.3%]).

**Conclusion:**

Perioperative bleeding outcomes are defined and analyzed with high inconsistency. Standardized outcome definitions and reporting practices are needed in this field to improve trial comparability and clinical relevance.

**Supplementary Information:**

The online version contains supplementary material available at https://doi.org/10.1186/s13741-026-00715-z.

## Background

More than 300 million people undergo surgery annually (Weiser et al. 2015), and perioperative mortality is estimated to range between 1 and 4% (Park et al. 2025; Devereaux et al. 2022). Major bleeding is the most common attributable cause of postoperative death, accounting for over 21% of surgical mortality, which exceeds sepsis and myocardial injury (Park et al. 2025). Despite its clinical importance and frequent use as an outcome in randomized controlled trials (RCTs) in surgery, the definition, measurement, and analysis of bleeding are not trivial tasks (Bartoszko et al. 2017). Trials may use continuous measures (e.g., quantified blood loss in millilitres or change in hemoglobin) (Piette et al. 2024), binary outcomes (e.g., blood transfusion rate) (Karanicolas et al. 2024; Martel et al. 2025), ordinal scales (e.g., visual clarity of the surgical site due to bleeding) (Kunze et al. 2025), or composite endpoints (e.g., combined major or life-threatening bleeding events) (Devereaux et al. 2022).

These differing approaches can affect the ability to detect meaningful treatment effects (Heneghan 2017), and have important implications for sample size requirements, optimal analytic strategies, and the interpretation of treatment effects (Campbell et al. 1995; Othus et al. 2022). For example, continuous surrogates are often statistically efficient and may require smaller sample sizes (Ragland 1992; Bhandari et al. 2002), but they can be harder to interpret clinically (Mayer 2019). In contrast, binary endpoints, such as transfusion rate, are more directly relevant to clinical and policy decision-making, though they tend to be statistically less efficient (Morton et al. 2018).

Improving how perioperative bleeding outcomes are defined and reported first requires a clear understanding of current practice. This review therefore summarizes current approaches in RCTs. The findings aim to inform future development of standardized definitions and analytic methods to improve clinical interpretability and cross-trial comparability.

## Methods

### Protocol registration

We registered this study with the International Prospective Register of Systematic Reviews (PROSPERO; registration ID: CRD420250607319) on March 19, 2025. The full protocol with amendments is available at: https://www.crd.york.ac.uk/PROSPERO/view/CRD420250607319. Reporting of this review follows the Preferred Reporting Items for Systematic Reviews and Meta-Analyses (PRISMA 2020) checklist (Tricco et al. 2018).

### Research questions

This review aimed to answer the following questions:

1. How are perioperative bleeding outcomes defined and measured in contemporary RCTs involving surgical patients?
2. How do bleeding outcome definitions vary according to study characteristics, including surgical specialty, intervention type, sample size, data type, and protocol-specified outcome status?
3. What statistical analysis and reporting approaches are used for perioperative bleeding outcomes?
4. How are sample size calculations and assumed effect sizes justified?
5. How do authors interpret treatment effects for perioperative bleeding outcomes?

### Eligibility criteria

We included the primary results publications of RCTs in the perioperative settings that evaluated any pharmacological or procedural interventions (such as surgical techniques or devices), where the primary outcome was related to perioperative bleeding. We limited inclusion to studies published between January 1, 2024, and April 9, 2025, without language restrictions, to capture the full year preceding the search. We chose a time-based restriction rather than restrictions based on journal impact or trial size to obtain a broad, contemporary, and representative sample across diverse methodologies and clinical settings.

We defined surgery as any invasive procedure involving tissue incision, dissection, or manipulation, whether performed via open, laparoscopic, or robotic approaches, in a controlled clinical setting. We excluded non-interventional endoscopic procedures (e.g., gastroscopy or colonoscopy only), percutaneous catheter-based interventions (e.g., percutaneous coronary interventions), image-guided radiologic procedures without therapeutic tissue removal, and non-surgical obstetric/gynecologic events (e.g., vaginal delivery). In this review, our focus is not limited to trials evaluating surgeries but rather to trials involving surgical patients. The literature categorizes trials into three types: 1) medical management; 2) comparisons of surgical techniques; and 3) surgical vs. non-surgical techniques (Ramchurn And Nundy 2024). The studies in the latter category were excluded.

Perioperative bleeding was defined as any bleeding, assessed either clinically (e.g., death to bleeding or re-operation) or through surrogate measures (e.g., blood loss in millilitres), occurring immediately after randomization up to the time defined by the study authors. We identified the primary outcome using the following hierarchy: authors explicitly labelled it as primary, we inferred it from the main objective or title, or it was the outcome used to determine the sample size of the study. If we could not determine the primary outcome, we excluded the study (Figure S1, Additional File 1).

### Search strategy

We conducted a comprehensive search in the Cochrane Central Register of Controlled Trials (CENTRAL) using a combination of keywords and MeSH terms (Table S1, Additional File 1). CENTRAL includes randomized trials from PubMed, Embase, CINAHL, ClinicalTrials.gov, and the World Health Organization International Clinical Trials Registry Platform. The final search was conducted on April 9, 2025.

### Study selection

We used Covidence (Covidence 2024) for study management and screening. At least two reviewers (YMK and NMZ or ME) independently screened titles and abstracts, followed by full-text screening. A calibration exercise was conducted on 10% of the included studies at the full-text review stage to ensure consistency in applying the eligibility criteria. Discrepancies were resolved through discussion among the reviewers or, when necessary, with a third reviewer (PSR).

### Data extraction

One reviewer (YMK) extracted the data in a standardized data extraction form, and a second reviewer (NMZ or ME) independently verified its accuracy. We contacted study authors and consulted trial registries for additional information where needed.

The following trial characteristics were extracted: study type (main trial or pilot), statistical objective (superiority, non-inferiority, or equivalence), population type (adult or pediatric), total sample size, surgical specialty, and type of comparison.

For each primary outcome, we extracted the definition, timing of assessment, type of data, details of statistical analyses, and reporting of treatment effect estimates and statistical inference. We also documented the studies’ justification for the assumed effect size used in sample size calculation. In addition, we extracted the exact wording used by the study authors in the discussion or conclusion to interpret their findings.

### Quality assessment

We were not concerned with estimates of treatment effects in this review, but rather with the methodological handling of study outcomes related to perioperative bleeding. Therefore, we did not assess the risk of bias that might arise from deficiencies in allocation and its concealment, blinding, or adherence to the intended intervention. However, we did take several quality elements into account. First, we focused only on randomized trials. Second, we excluded studies in which the primary outcomes were not specified. Third, we stratified analyses of the primary objective of this review by sample size and by whether the primary outcome was reported consistently with a prospectively registered or published protocol.

### Data synthesis/analysis

To address the primary objective, we prepared a complete list of definitions used for perioperative bleeding as a primary outcome and categorized them into conceptually similar domains. For each domain, we summarized the frequencies and the specific definitions, including the timing of outcome assessments. We plotted the distribution of outcome domains across surgery type, intervention type, data type, sample size quartiles, and protocol-specified outcome status using heatmaps and dot plots, where appropriate.

For secondary objectives, we summarized how often studies conducted a sample size calculation based on the primary endpoint and how the assumed effect size was justified. We also summarized statistical analysis approaches and reporting practices. Finally, we categorized how authors interpreted their findings in the discussion or conclusion sections, using inductive grouping into similar interpretative approaches. This was done separately for superiority and non-inferiority/equivalence trials. We then reported the frequencies for each interpretation category.

## Results

**Fig. 1:**
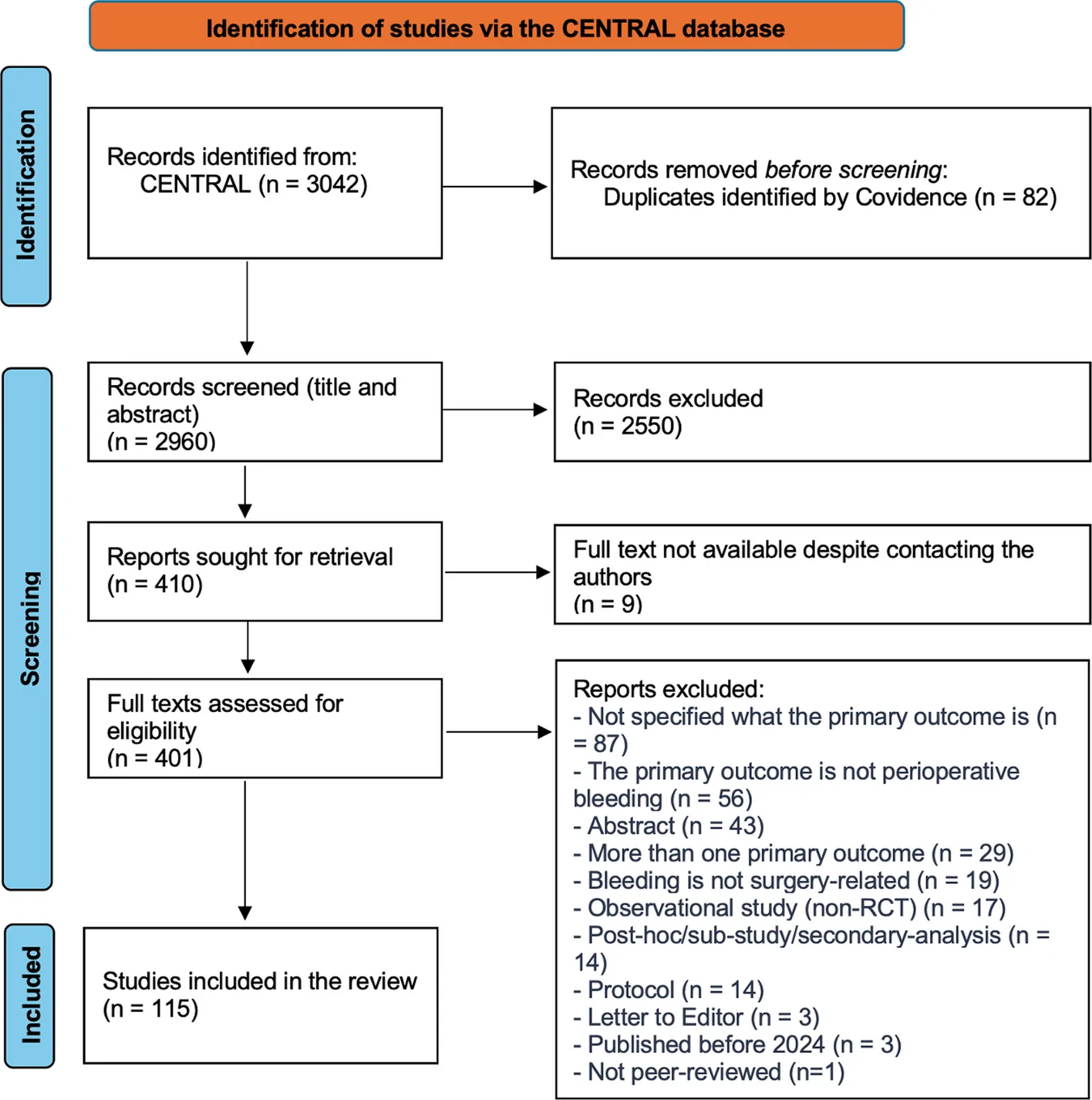
PRISMA flowchart

We screened 2,960 unique records and excluded 2,559 at the title/abstract stage. Of the 401 records screened at the full-text stage, 115 were eligible. Reasons for exclusion at this stage are detailed in the PRISMA flow diagram in Fig. 1. The most common reason for exclusion was the lack of a clearly specified primary outcome (*n* = 87).

### Study characteristics

Table 1 shows the characteristics of the included studies. Most studies were small, with 43.5% enrolling fewer than 100 participants. Most (89.6%) were superiority trials, and almost all (96.5%) randomized individual patients with a parallel-arm design. The most common surgical category was orthopedic (25.2%), followed by ENT (ear, nose, and throat)/head and neck (16.5%) and gynecologic surgery (16.5%). Most trials (76.5%) evaluated pharmacological interventions, with half of those (*n* = 46 [52.3%]) involving tranexamic acid. Additional File 2 provides the full details extracted from the included studies.

**Table 1:** Characteristics of the included studies (*N* = 115)

Characteristic	Category	Frequency n (%)
**Study type**	Full (non-pilot)	109 (94.8)
	Pilot	6 (5.2)
**Study objective**	Superiority	103 (89.6)
	No-inferiority	11 (9.6)
	Equivalence	1 (0.9)
**Trial architecture**	Parallel-arm design	111 (96.5)
	Split-body design	4 (3.5)
**Population**	Adults	108 (93.9)
	Children	4 (3.5)
	Adults and Children	3 (2.6)
**Total sample size**	< 100	50 (43.5)
	100–199	42 (36.5)
	200–299	14 (12.2)
	≥ 300–399	9 (7.8)
**Type of surgery**	Orthopaedic	29 (25.2)
	ENT/head and neck	19 (16.5)
	Gynecologic	19 (16.5)
	General	12 (10.4)
	Urologic	10 (8.7)
	Plastic/reconstructive	8 (7.0)
	Cardiac	7 (6.1)
	Mixed	6 (5.2)
	Ophthalmologic	3 (2.6)
	Neurologic	2 (1.7)
Interventions evaluated*	**Pharmacological**	**88 (76.5)**
	Pharmacologic intervention vs placebo	36 (31.3)
	Pharmacologic intervention vs its absence	18 (15.6)
	Different pharmacologic interventions (± control)	16 (13.9)
	Different routes/forms of administration of a pharmacologic intervention (± control)	9 (7.8)
	Different dosages of same pharmacologic intervention (± control)	9 (7.8)
	**Procedural**	**27 (23.5)**
	Different surgical techniques	22 (19.1)
	Device or technique vs no device/technique	5 (4.3)

^*^Interventions were categorized according to their primary mechanism of action. Pharmacological interventions involved drug-based therapies affecting coagulation or hemostasis, and procedural interventions involved surgical techniques, devices, or mechanical manoeuvres

### Perioperative bleeding definitions and measurements

As summarized in Table 2, among the 116 bleeding outcome definitions reported across 115 RCTs (one study met the inclusion criteria with two primary outcomes) (Kim and Park 2025), the most common approach was volumetric and gravimetric measures (39.6%), typically based on suction volume, drain output, weighing of absorbent materials, or combinations of these. Calculated or formula-based estimates were the second most common (19.0%) domain. Clinician/observer-reported outcomes accounted for 20 definitions (17.2%) and largely reflected visual bleeding severity scales or measures of time to, or success rate of, hemostasis. Eleven studies (9.5%) used blood transfusion-based metrics, such as transfusion rate or number of units transfused. Less frequently reported were hemoglobin or hematocrit change (6.9%), patient-reported bleeding events (4.3%), and clinical management-based outcomes (e.g., re-intervention) (3.4%). Table S2 in Additional File 3 shows how the timing of assessment varied between and within definitions. Bleeding was most assessed during surgery (44.8%), followed by within the first week postoperatively (21.6%) (Figure S2, Additional File 3).

**Table 2:** Definitions of perioperative bleeding outcome (*N* = 116)

Domain	Frequency n (%)	Outcome definition
Volumetric and gravimetric measures	46 (39.6)	Suction volume only* (Akbari et al. 2024; Allahyani et al. 2024; Park et al. 2024; Sharifi et al. 2024; (Singh n.d.)
		Drain output† (Hosseini et al. 2024; Janowski et al. 2025; Mikić et al. 2024; Singh et al. 2024; Verma et al. 2024; Zhou et al. 2024; Fahmy And Yasin 2024)
		Weight of absorbent materials‡ (Chaichumporn et al. 2024; Colclough et al. 2024; Tejada et al. 2024)
		Suction volume + Drain output (Piette et al. 2024)
		Suction volume + Weight of absorbent materials (objectively assessed) (Abdelsalam 2024); Ansari et al. 2024; Arvind et al. 2025; Baghdasaryan et al. 2024; Chen et al. 2025; Elmizadeh et al. 2024; Heydari et al. 2024; Huang et al. 2025; Imaralu et al. 2024; Lv et al. 2024; Masoumzadeh et al. 2025; Meng et al. 2025; 2024; Nasief et al. 2024; Naz et al. 2024; (Srilekha 2024); Sumphaongern And Chantarangsu 2025; Xu et al. 2024; Yl et al. 2024; (Zaman 2024); Sinha et al. 2024)
		Suction volume + Estimated blood absorbed by absorbent materials (subjectively assessed) (Adibparsa et al. 2024; Calim et al. 2024; (Kamali 2024); Kargar-soleimanabad et al. 2024; Vaezi et al. 2025)
		Suction volume + Weight of absorbent materials + Drain output (Abdelsami Aiad et al. 2024; Elbaseet et al. 2025; Olaleye et al. 2024)
		Number of absorbent pads used (Kovalis et al. 2024)
Calculated or formula-based estimates	22 (19.0)	Hct-based formula (Gross Formula)§ (Alasaad 2024); Atef et al. 2024; Güngördük et al. 2025; Han et al. 2024; Krauss et al. 2024; Li et al. 2025; Lou et al. 2024; Neumann et al. 2024; Ye et al. 2024; Ye et al. 2025)
		Hb-based formula (Bosilah et al. 2024; Hosseini-Monfared et al. 2025)
		Irrigation-based Hb formula‖ (Rohith et al. 2024)
		(Gross formula – DBL**) + Allogenic blood transfusion (Dong et al. 2024; Luo et al. 2024)
		Gross formula + Transfused blood (Luo et al. 2025; Naderi et al. 2025; Xie et al. 2024)
		Total Hb loss (%)†† (Chaiyakit et al. 2024; Miralles-Muñoz et al. 2024; Pinsornsak et al. 2024)
Clinician/observer-reported measures	20 (17.2)	Wormald scale§§ (Abdelaziz et al. 2024)
		Fromme–Boezaart scale‡‡ (Ghazaly et al. 2024; Mohamed Hassan et al. 2024; Singh et al. 2024; Vaghardoost et al. 2024; Zhang et al. 2024)
		Modena bleeding score (Zhang et al. 2024)
		Author-developed 3-Point visual field clarity scale (Kunze et al. 2025)
		Hemostasis achieved‖‖ (An et al. 2024; Balanescu et al. 2024; House et al. 2024; Luo et al. 2024; Shady et al. 2024)
		Time in minutes or seconds to achieve bleeding control (Kang et al. 2025; Kenny et al. 2024)
		Author-defined 4-point scale assessing blood loss and transfusion need (Sarode et al. 2024)
		Clinically detected bleeding during postoperative evaluation (Su et al. 2024)
		Visual severity or extent of ecchymosis or hematoma (rated by scale or measured area) (Cano Valls et al. 2024; Marous et al. 2024; Paramo et al. 2024)
Blood transfusion-based metrics	11 (9.5)	Transfusion rate (whether at least one transfusion was administered) (Karanicolas et al. 2024; Martel et al. 2025; Breau et al. 2024; Gao et al. 2024; Khatib et al. 2024; Mastroianni et al. 2024; Saour et al. 2024)
		Volume of blood transfused (Ismail And Mahrous 2024; Ravichandran et al. 2025)
		Number of units transfused (Mahdy et al. 2025)
		Composite of transfusion > 2 RBC units or postoperative infection (Kiviniemi et al. 2024)
Hemoglobin or hematocrits change	8 (6.9)	Difference between pre- and postoperative Hb or Hct levels (Kundargi et al. 2024; Langar et al. 2025; Lee et al. 2025; McDermott et al. 2025; Nouralizadeh et al. 2025; Omar et al. 2024)
		Hb concentration in irrigation fluid*** (Diab et al. 2024)
		Rate of deviation from standard Hb or Hct concentrations (Demirpolat et al. 2024; Park et al. 2024)
Patient-reported bleeding events	5 (4.3)	Bleeding symptoms reported directly by the patient (Ali et al. n.d.); Radtke et al. 2024)
		Patient-reported bleeding duration (in days) (Kim and Park 2025)
		Incidence of hematoma (presence/absence in clinical examination) (Yao et al. 2024; Yeow et al. 2025)
Clinical management of blood loss	4 (3.4)	Need for additional treatment other than the main intervention (Kim and Park 2025)
		Need for re-intervention or hospitalisation (Chiang et al. 2025)
		Universal Definition of Perioperative Bleeding††† (Bratt et al. 2024)
		Clinically Significant Delayed Bleeding‡‡‡ (Drews et al. 2025)

*Abbreviations*: *DBL* Dominant blood loss, *EBV* Estimated blood volume, *Hb* Hemoglobin, *Hct* Hematocrit, *RBC* Red blood cells

^*^Blood collected in suction canisters, with or without adjustment for irrigation fluid

^†^Volume of blood collected from surgical drains up to various postoperative time points

^‡^Gauze, sponges, or dressings weighed before and after use

^§^Estimated blood volume (EBV) × [(preoperative Hct – postoperative Hct)/preoperative Hct]

^¶^ [(Initial Hb − postoperative Hb)/average Hb] × EBV

‖ [(Hb in fluid × irrigation volume × 1000)/(preoperative Hb × 5.2)]

^**^ DBL = (weight of soaked gauze − dry weight) + (suction volume − irrigation volume)

^††^ [(Hb loss/initial Hb) × 100], where Hb loss = blood volume × (initial Hb − postoperative Hb) + Hb transfused

^‡‡^ An 11-point (0–10) grading scale used to evaluate surgical field quality during endoscopic sinus surgery

^§§^ A 6-point (0–5) grading scale used to assess the severity of intraoperative bleeding based on the frequency of suctioning required

^¶¶^ A 5-point (1–5) scale used to quantify the severity of intraoperative bleeding

‖‖ Whether bleeding stopped within a specified time after application of the intervention, as subjectively assessed

^***^ Spectrophotometrically measured Hb concentration in irrigation fluid (absorbance of fluid × 367.7)

^†††^ Includes criteria such as delayed sternal closure, > 1000 mL bleeding, > 4 units transfused, factor VII use, or surgical re-exploration

^‡‡‡^ Defined as post-procedure bleeding leading to transfusion, reintervention, or other hospital-based clinical events

Note: References correspond to the included studies in which each definition was used

### Bleeding outcome definitions by study characteristics

**Fig. 2:**
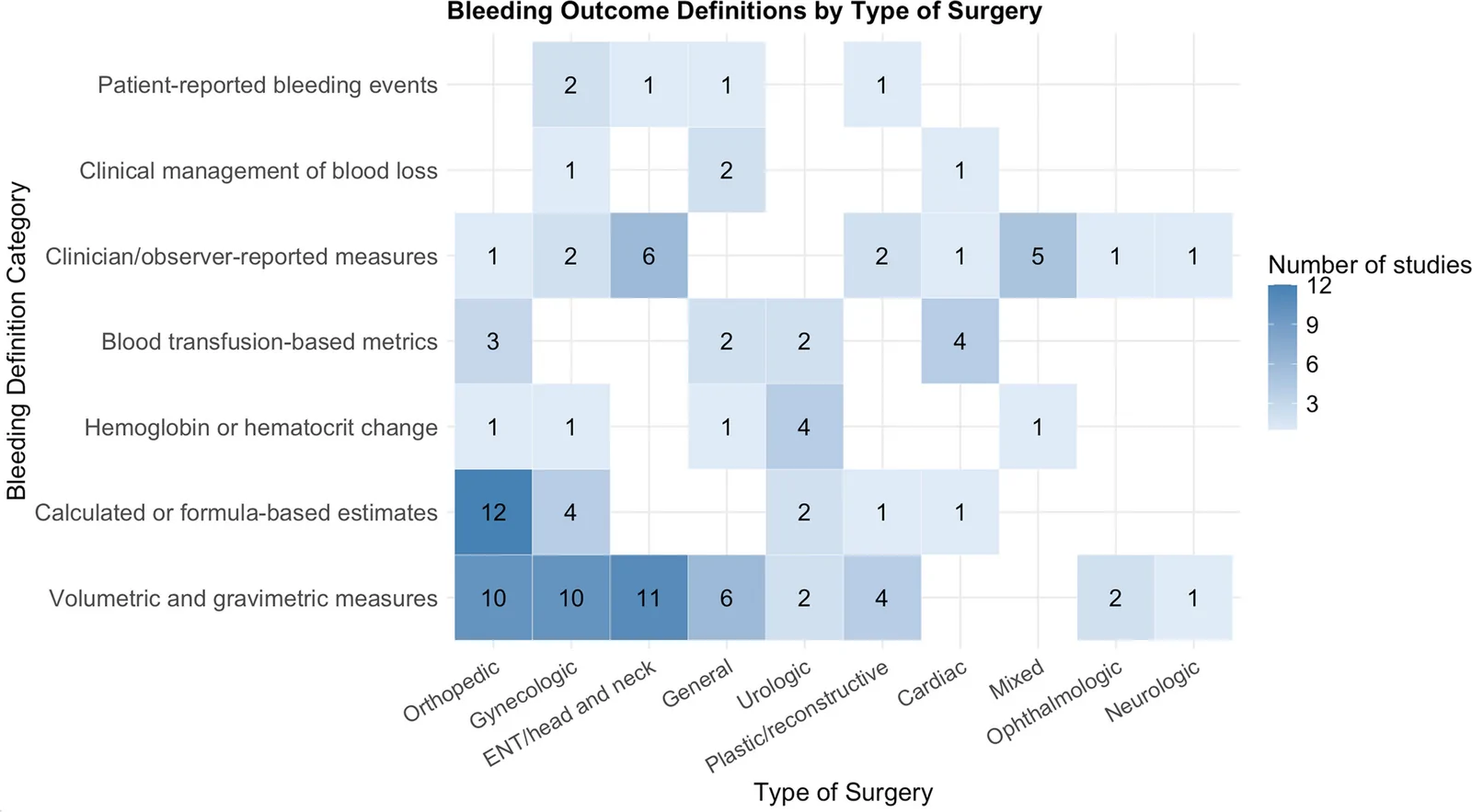
Distribution of bleeding outcome definitions across surgery types (*N* = 116)

The distribution of bleeding outcome domains by surgical specialty is shown in Fig. 2. Volumetric and gravimetric measures predominated across most surgery types. Blood transfusion-based metrics were most frequent in cardiac surgery, while clinician/observer-reported measures were common in ENT/head and neck and mixed-procedure studies. Patient-reported events and clinical management measures were most frequently observed in gynecologic and general surgery trials, respectively.

Figures S3-S6 (Additional File 3) show outcome domains stratified by sample size, data type, protocol-specified outcome status, and intervention type. Most small studies used volumetric and gravimetric measures, whereas patient-reported, clinical management, and blood transfusion-based metrics were not common in smaller trials; this imbalance was less pronounced among larger studies. Patient-reported, clinical management, and blood transfusion-based metrics were also more frequently reported in studies with a protocol-specified primary outcome than in those without. Across most domains, pharmacological intervention trials outnumbered trials with procedural intervention; this imbalance was most pronounced for clinical/observer-reported measures.

### Sample size considerations

As shown in Table 3, 90 of 115 studies (78.3%) reported conducting a sample size calculation based on the primary outcome. Seventeen studies (14.8%) did not report a sample size calculation, two (1.7%) based the calculation on a non-primary outcome, and six (5.2%) did not specify the outcome used.

**Table 3:** Sample size considerations, analysis and reporting practices

Characteristics	Category	Frequency n (%)
**Sample size considerations**		
Pre-study sample size calculation based on the primary outcome*	Yes	90 (78.3)
	No	25 (36.7)
**Justification for assumed effect size (*n* = 90)**	No Justification	34 (37.8)
	Effect size from a previous primary study (not a pilot study for the current trial)	26 (28.9)
	A clinically important difference	10 (11.1)
	Effect size derived from the pilot phase of the trial	8 (8.9)
	A standardized effect size (e.g., Cohen’s d)	6 (6.7)
	Effect sizes derived from the internal institutional data	1 (1.1)
	No information on the assumed effect size	5 (5.6)
**Analysis approaches**		
**Type of data**†	Continuous	77 (66.4)
	Binary	19 (16.4)
	Ordinal	13 (11.2)
	Count	3 (2.6)
	Composite	3 (2.6)
	Time-to-event	1 (0.9)
**Type of test**†	Parametric	69 (59.5)
	Non-parametric	36 (31.0)
	Both	6 (5.2)
	N/A‡	5 (4.3)
Adjustment for baseline covariates*	Yes	18 (15.6)
	No	97 (84.3)
**Reporting approaches**		
Effect estimate reported*	Yes	34 (29.6)
	No	81 (70.4)
Confidence interval reported*	Yes	28 (24.3)
	No	87 (75.6)

^*^Denominator is the number of studies (*N* = 115)

^†^Denominator is the number of outcomes (*N* = 116)

^‡^Not reported, no test was done, or Bayesian analysis

The most common justification for the assumed effect size was evidence from prior studies (28.9%). Of the 10 studies (11.1%) which defined the assumed effect size as clinically important, only one study reported using expert consultation to define a minimal clinically important difference (Chen et al. 2025); others did not provide a clear rationale or supporting evidence.

### Analysis and reporting practices

Most bleeding outcomes were analyzed as continuous variables (66.4%). Two studies (Sarode et al. 2024; Su et al. 2024) dichotomized their ordinal outcomes at subjective thresholds, and two studies transformed their ordinal scale into a continuous outcome after multiple measures at different time points (Kunze et al. 2025; Zhang et al. 2024). Non-parametric tests were used exclusively in 36 analyses (31.0%), and only 18 studies (15.6%) adjusted for baseline covariates in their primary analysis. Effect estimates were reported in 34 studies (29.6%), and confidence intervals in 28 (24.3%).

Table S3 (Additional File 3) presents sample sizes and analysis practices by outcome type. Median sample sizes were lowest for count (50 [25th–75th percentile: 32–140]), ordinal (96 [70–130]), and continuous outcomes (100 [60–150]), and highest for binary outcomes (180 [128–353]). Binary outcomes were more likely than other types to report effect estimates. All three composite outcomes were analyzed using non-parametric chi-square tests, with no effect estimates reported.

### Interpretation of treatment effects

Table 4 summarizes how treatment effects were interpreted in the discussion or conclusion sections of the included studies. In superiority trials, the most common approach was referencing statistical significance alone, occasionally with the direction of effect (e.g., “significantly better”). Of the studies addressing clinical significance, some acknowledged uncertainty despite statistical significance (Ghazaly et al. 2024; Demirpolat et al. 2024; Park et al. 2024), while others claimed clinical importance without defining a threshold (Meng et al. 2025; Sumphaongern And Chantarangsu 2025; McDermott et al. 2025). Among the 12 non-inferiority and equivalence trials, none referred to their pre-specified margins in the discussion or conclusion, and only one study included confidence intervals around their effect estimate.

**Table 4:** Summary of how treatment effects were interpreted in included trials (*N* = 115)

Study Objective	Interpretation Category	Description	Examples	Frequency n (%)
**Superiority (*n* = 103)**	**Binary interpretation** (Karanicolas et al. 2024; Akbari et al. 2024; Park et al. 2024; Singh n.d.; Mikić et al. 2024; Elmizadeh et al. 2024; Kamali 2024; Güngördük et al. 2025; Han et al. 2024; Ye et al. 2024; Pinsornsak et al. 2024; Zhang et al. 2024; Shady et al. 2024; Cano Valls et al. 2024; Breau et al. 2024; Ravichandran et al. 2025; Kiviniemi et al. 2024; Lee et al. 2025; Nouralizadeh et al. 2025; Omar et al. 2024; Bratt et al. 2024)	Used qualitative terms (e.g., “reduced,” “no change”) without providing statistical or clinical justification	*- “bleeding in the remifentanil group was less than the other two groups”* *- “This trial demonstrated no improvement in blood transfusion.”*	21 (20.4)
	**Statistical significance only ** Kunze et al. 2025; Kim and Park 2025; Hosseini et al. 2024; Verma et al. 2024; Zhou et al. 2024; Fahmy And Yasin 2024; Chaichumporn et al. 2024; Heydari et al. 2024; Huang et al. 2025; Imaralu et al. 2024; Lv et al. 2024; Masoumzadeh et al. 2025; Naz et al. 2024; Alasaad 2024; Yl et al. 2024; Zaman 2024; Sinha et al. 2024; Adibparsa et al. 2024; Kargar-soleimanabad et al. 2024; Vaezi et al. 2025; Abdelsami Aiad et al. 2024; Elbaseet et al. 2025; Olaleye et al. 2024; Kovalis et al. 2024; Li et al. 2025; Neumann et al. 2024; Rohith et al. 2024; Dong et al. 2024; Luo et al. 2024; Luo et al. 2025; Naderi et al. 2025; Xie et al. 2024; Chaiyakit et al. 2024; Miralles-Muñoz et al. 2024; Abdelaziz et al. 2024; Mohamed Hassan et al. 2024; Vaghardoost et al. 2024; Kang et al. 2025; Su et al. 2024; Marous et al. 2024; Paramo et al. 2024; Gao et al. 2024; Ismail And Mahrous 2024; Mahdy et al. 2025; Diab et al. 2024; Ali et al. n.d.; Yao et al. 2024; Yeow et al. 2025; Chiang et al. 2025)	Interpretation based solely on statistical significance (e.g., *p*-values or phrases like “significant”) without numerical estimates or clinical context	*- “We found a significant decrease in blood loss in those who received TXA”* *- “The milrinone group showed less blood loss and better surgical field grading than did the nitroglycerin group (P* < *0.05).”*	49 (47.6)
	**Data only (no clinical interpretation) ** Allahyani et al. 2024; Sharifi et al. 2024; Janowski et al. 2025; Singh et al. 2024; Tejada et al. 2024; Ansari et al. 2024; Arvind et al. 2025; Baghdasaryan et al. 2024; 2024; Nasief et al. 2024; Calim et al. 2024; Atef et al. 2024; Lou et al. 2024; Ye et al. 2025; Bosilah et al. 2024; Hosseini-Monfared et al. 2025; Singh et al. 2024; Kenny et al. 2024; Mastroianni et al. 2024; Saour et al. 2024; Kundargi et al. 2024; Langar et al. 2025; Drews et al. 2025)	Reported group-level values or effect estimates with or without statistical significance, but no further interpretation or clinical relevance	*- “The mean intraoperative blood loss was 124.1* ± *34.85 mL in the NP group and 115.63* ± *31.89 mL in the IV group. …, the difference was not statistically significant.”*	23 (22.3)
	**Reference to clinical significance** (Martel et al. 2025; Colclough et al. 2024; Meng et al. 2025; Sumphaongern And Chantarangsu 2025; 2024; Ghazaly et al. 2024; Khatib et al. 2024; McDermott et al. 2025; Demirpolat et al. 2024; Park et al. 2024)	Interpretation included implicit or explicit discussion of whether the results were clinically meaningful	*- Implicit: “Intraoperative blood loss volume as the primary outcome difference between the groups is not clinically significant despite reaching statistical significance”* *- Explicit: “An *a priori* power calculation was conducted to detect a clinically important difference of 20% reduction in blood transfusion. The results showed a significant reduction (40%, p* = *0.04) in the number of patients who required a transfusion.”*	10 (9.7)
**Non-inferiority and Equivalence (*n* = 12)**	**Non-inferiority claims only ** Piette et al. 2024; An et al. 2024; House et al. 2024; Sarode et al. 2024)	Simply stated “non-inferior” without further numerical support	*- “A thrombin-based hemostatic agent is noninferior to Floseal.”*	4 (33.3)
	**Non-inferiority claim with limited support** (Chen et al. 2025; Xu et al. 2024; Luo et al. 2024)	Included arm-specific values, effect estimates, or statistical significance in addition to non-inferiority claim	*- “the clinical effectiveness of Borayflo Haemostatic matrix was found to be non-inferior to that of Surgiflo Haemostatic matrix, with rates of 93.14% and 94.89% respectively.”*	3 (25.0)
	**Non-inferiority claim with confidence intervals** (Balanescu et al. 2024)	Included confidence intervals to the effect estimate in support to the non-inferiority claim	*- “The percentage of patients achieving hemostasis was 96.7% in the FS Grifols treatment group and 96.4% in the Evicel treatment group. The relative RR was 1.01 (95% CI 0.96—1.07) showing that FS Grifols was non-inferior to Evicel.”*	1 (8.3)
	**No clear non-inferiority/equivalence interpretation** (Abdelsalam 2024; Krauss et al. 2024; Zhang et al. 2024; Radtke et al. 2024)	Did not claim non-inferiority or equivalence; instead, presented statistical significance, arm-specific data, or concluded clinical significance without predefined threshold	*- “Our study found a significant reduction in patients’ perception of PVB when the intervention was performed.”*	4 (33.3)

## Discussion

This review summarizes how perioperative bleeding is defined, analyzed, reported, and interpreted in 115 randomized controlled trials. Our findings showed high variability in definitions and some methodological weaknesses.

### Perioperative bleeding definitions and measurements

Our findings support existing literature, which highlights key challenges in RCT outcome reporting for perioperative clinical trials, including variability in outcomes and the specific endpoints reported across trials, inconsistency in their measurement, questionable measurement quality, and scarcity of patient-relevant outcomes (Tunis et al. 2016).

In just 115 trials, we identified 36 unique definitions for perioperative bleeding primary outcomes resulting from different constructs and methods of ascertainment. This raises the question of which outcome best reflects clinical relevance and should be prioritized to inform practice (Gargon et al. 2011). The lack of a standardized definition in this field contrasts with more widely accepted bleeding definitions across other interventional fields. The Bleeding Academic Research Consortium (BARC), for example, developed widely adopted definitions for cardiovascular trials that standardized reporting across studies of antithrombotic therapy and device interventions (Mehran et al. 2011). Similarly, the National Heart, Lung, and Blood Institute and the U.S. Department of Defense have issued recommendations for primary outcomes in trials of hemostatic interventions across various domains (Spinella 2021), and the International Society of Thrombosis and Haemostasis (ISTH) (Khorsand et al. 2021) has provided guidance on defining hemostatic effectiveness in major bleeding. However, these initiatives are not directly applicable to perioperative bleeding, as most bleeding-focused clinical trials concentrate on the effectiveness of interventions targeting bleeding specifically related to the procedure, rather than bleeding from any site. Therefore, perioperative bleeding may benefit from a field-specific standardization of outcome definitions.

Questionable measurement validity and reliability were another challenge. Although prior literature has noted the inaccuracies associated with gravimetric, volumetric, and formula-based methods of blood loss estimation (Gerdessen et al. 2021), more than half of the outcomes in our review used these approaches. Transfusion-related outcomes are also criticized due to their reliance on clinician judgment or guideline-driven protocols, which vary widely across institutions (Baker et al. 2021). Of the 10 transfusion-related outcomes in our sample, five were based on subjective decision-making, while the others used protocolized hemoglobin thresholds that differed across studies.

Another challenge was the frequent use of outcomes with limited patient relevance. Although no published studies have identified which perioperative bleeding outcomes matter most to patients, those generally considered important, such as mortality, need for reoperation, rehospitalization, or major complications (Møller 2019), were rarely used as primary endpoints in the included studies. Surrogate outcomes such as volumetric or gravimetric blood loss in millilitres are not easy to interpret from a patient’s perspective. Despite their practicality, surrogates are usually continuous measures and therefore allow smaller sample sizes (Piantadosi 2024; Weintraub et al. 2015; Velentgas 2013), which makes clinical outcomes (often secondary or safety) harder to interpret. Surrogates also offer limited support for clinical decision-making and regulatory approval. According to the U.S. Food and Drug Administration (Research Center for Drug evalutaion and Research 2025; Research Center for Drug evalutaion and Research 2025), trials should use outcomes that are standardized, meaningful, and developed through a transparent process. Without alignment with patient priorities, trial outcomes are less likely to gain regulatory acceptance and may contribute to research waste.

Finally, the timing of assessment also varied widely (intraoperatively to 30 days after surgery). Measuring the same outcome at different times can give different impressions of the intervention’s effect (Feely 2020). Although definitions mainly dictate when bleeding is measured, even within a single definition (e.g., calculated blood loss or need for transfusion), assessments can occur at different time points. Moreover, assessment timing should capture the period during which the outcome is most likely to occur (Halme et al. 2024). A cohort study found that most major bleeding events occur within the first week after surgery (Halme et al. 2024). This information helps ensure follow-up is neither unnecessarily long (wasting resources) nor too short, which could miss relevant events.

### Sample size considerations

It is recommended that the assumed effect sizes used in sample size calculations reflect realistic and clinically important differences (Parker 2023; Schulz And Grimes 2005; Hislop et al. 2014). However, only 9% of the included studies stated using a clinical threshold, and only one explained how that threshold was determined. Unlike other surgical complications, such as pain, quality of life, and functional activity, where some efforts have been made to define minimally important differences (MID) (Danoff et al. 2018; Magouliotis et al. 2023; Rice et al. 2024), no such standardized thresholds currently exist for perioperative bleeding outcomes. This likely reflects the lack of evidence on what constitutes a patient-important bleeding effect. Unlike outcomes such as pain or quality of life, bleeding is typically measured indirectly from the patient’s perspective (Zajak 2024), which makes it difficult to apply anchor-based methods that rely on patient-reported outcomes to establish meaningful change. In addition, the absence of consensus-based methods and the high variability in institutional protocols and clinician judgment further limit the development of standardized MIDs in this area (Baker et al. 2021).

More than a third of studies based their effect estimate on results from their pilot study or another primary study. While previous and pilot studies can provide useful inputs for parameters such as standard deviations or baseline prevalence, the appropriateness of using their effect estimates is debated. Some caution that effect estimates from existing studies merely show observed differences rather than those that are clinically important (Parker 2023; Dettori et al. 2022). In addition, pilot studies are often underpowered and provide unstable estimates that may over- or underestimate the true effect, which can in turn lead to underpowered or unnecessarily large main trials (Kistin And Silverstein 2015). The Difference ELicitation in TriAls Systematic Review noted that no single best method exists for specifying target differences and recommended transparency about whether the estimate reflects a clinically important, realistic, or both types of difference (Hislop et al. 2014). Our findings showed that this level of transparency remains uncommon in trials of perioperative bleeding outcomes.

### Analysis and reporting practices

This review showed heterogeneity in perioperative bleeding outcome data types, the corresponding analytic approaches, and reporting practices. Continuous outcomes were the most commonly used type, likely due to their greater statistical power and lower sample size requirements (Campbell et al. 1995). This was reflected in our findings, where trials using continuous measures had substantially smaller median sample sizes than those using binary outcomes. However, this efficiency comes with a trade-off; continuous outcomes may be less intuitive and harder for clinicians and patients to interpret (Mayer 2019).

Statistical analysis methods often did not match the data type. More than 60% of outcomes were analyzed using parametric tests, including several ordinal outcomes where assumptions of normality are likely violated (Phrase Complesion Scales 2005). Although non-parametric tests are more appropriate in these situations, they typically do not provide straightforward effect estimates or confidence intervals (Whitley And Ball 2002) recommended by CONSORT (Moher et al. 2010). Promising alternatives, such as proportional odds models (McCullagh 1980) and the win probability approach (Zou et al. 2023), can address these issues by preserving the ordinal nature of the data while providing interpretable effect measures. However, no trial in our review used these methods.

Only a minority of studies used regression models or stratified statistical methods that allow for adjustment of baseline covariates during the analysis stage, despite recommendations to do so (Holmberg And Andersen 2022). The low number of trials applying these methods shows a missed opportunity to improve precision (Holmberg And Andersen 2022). This is especially important in the context of perioperative bleeding, where numerous patient- and surgery-related factors have been identified as strong predictors of bleeding risk (Lopes et al. 2015; Tafur et al. 2020). Notably, none of the count, composite, or untransformed ordinal outcomes were analyzed using adjusted models, which likely reflects reliance on basic or inappropriate statistical methods for handling these types of data.

### Interpretation of treatment effects

Almost half of the superiority trials interpreted their findings based only on statistical significance, with some making strong recommendations for or against the use of an intervention based solely on statistically significant findings (Fahmy And Yasin 2024; Arvind et al. 2025). Statistical significance only shows whether an observed effect is unlikely to be due to chance and does not show the magnitude or importance of the effect (Goodman 2008). Some experts have advocated for moving away from statistical significance to effect size and confidence interval, and leaving the judgment to the reader (Bauchner et al. 2019), and some have emphasized the need to interpret findings against a predefined threshold of clinical importance (Thabane et al. 2022). However, few trials provided confidence intervals, and even fewer referenced clinical thresholds, likely due to the broader challenge of defining clinically meaningful values for perioperative bleeding outcomes.

Interpretation was more limited in non-inferiority and equivalence trials. Despite CONSORT recommendations to discuss pre-specified margins and present confidence intervals alongside effect estimates (Piaggio et al. 2012), none of the included trials discussed their non-inferiority or equivalence margins in the discussion or conclusion, and only one study reported confidence intervals to support their claim.

### Future directions

Consensus-based core outcome sets may improve standardization of perioperative bleeding outcomes. However, combining multiple bleeding outcomes into clinically meaningful and statistically efficient measures remains challenging. Composite outcomes were rarely used and often lacked justification for combining components of different importance. While composite outcomes can help capture the diverse manifestations of perioperative bleeding, especially given the variation in definitions observed in this review, it is important to consider the varying clinical importance of individual components (Dash et al. 2022). Hierarchical composite endpoints offer a way to integrate multiple dimensions of patient experience (benefits, harms, and clinical events) into a single interpretable measure (McCoy 2018; Gasparyan et al. 2022; Overbey et al. 2025). Despite their successful use in nephrology and cardiovascular trials (Packer 2016; Leifer 2025; Little et al. 2023), this type of endpoint remains absent from the perioperative bleeding field. By ranking outcomes according to clinical importance and prioritizing more meaningful events over device- or biomarker-based surrogates, hierarchical composite endpoints may better reflect patient-relevant benefit-harm trade-offs while preserving statistical efficiency (McCoy 2018; Gasparyan et al. 2022).

### Strengths and limitations

We reviewed a large sample of 115 contemporary trials, which allowed us to identify major patterns in several methodological aspects of perioperative bleeding outcomes, including their definition, analysis, reporting, and interpretation. These findings may serve as an important foundation for future efforts aimed at standardizing bleeding outcomes and improving methodological practices in this widely studied field. This review has several limitations. First, restricting the search to studies published over approximately one year may have resulted in omission of large or influential trials in the field. However, our objective was to obtain a broad and contemporary sample of current research practices across surgical specialties and intervention types, regardless of study size, rather than to provide a historical overview of bleeding outcome methodology. Second, our review does not address how bleeding outcomes were handled when assessed as secondary outcomes because we included only studies in which bleeding was a primary outcome. This focus was intentional, as primary outcomes most strongly influence trial design decisions, including sample size determination, statistical analysis, and interpretation of treatment effects.

## Conclusion

Perioperative bleeding is assessed in randomized trials using highly variable definitions, analytic methods, and interpretive frameworks. These methodological choices have important implications for trial design, inference, and comparability. Developing standardized, clinically meaningful bleeding outcomes and improving analytic and reporting practices are critical to strengthening evidence from perioperative trials.

## Supplementary Information

Additional file 1. Study selection guide, Search Strategy.

Additional file 2. Full data extracted from included studies.

Additional file 3. Timing of assessment, study characteristics by bleeding definition domains, and analysis practices by data type.

## Data Availability

No datasets were generated or analysed during the current study.

## Abbreviations

BARC: Bleeding Academic Research Consortium
CENTRAL: Cochrane Central Register of Controlled Trials
ENT: Ear Nose Throat
ISTH: International Society of Thrombosis and Haemostasis
MID: Minimally Important Difference
PRISMA: Preferred Reporting Items for Systematic Reviews and Meta-Analysis
PROSPERO: International Prospective Register of Systematic Reviews
RCT: Randomized Controlled Trial
WinP: Win Probability
