# Supplementary material for: Definition, analysis, reporting, and interpretation of perioperative bleeding in randomized controlled trials: a methodological systematic review
Source: Perioper Med (Lond). 2026 Jul 2;15:80. doi: 10.1186/s13741-026-00715-z (PMC13595797; doi:10.1186/s13741-026-00715-z)
Supplement: Supplementary file 1 [file 13741_2026_715_MOESM1_ESM.docx]

**Additional File 1**

**Figure S1. Decision guide for identifying the primary outcome**

**Table S1. Search strategy**

| Search Name: | Perioperative Bleeding |  |
| --- | --- | --- |
| Date Run: | 09/04/2025 20:51:51 |  |
| Comment: | New April 9th |  |
|  |  |  |
| ID | Search | Hits |
| #1 | MeSH descriptor: [General Surgery] explode all trees | 511 |
| #2 | MeSH descriptor: [Surgical Procedures, Operative] explode all trees | 172165 |
| #3 | surg* OR operat* OR  postsurg* OR postoperat* OR perioperat* OR intraoperat* | 457770 |
| #4 | #1 OR #2 OR #3 | 502354 |
| #5 | MeSH descriptor: [Hemorrhage] explode all trees | 20484 |
| #6 | MeSH descriptor: [Blood Transfusion] explode all trees | 4695 |
| #7 | MeSH descriptor: [Hemostasis] explode all trees | 5950 |
| #8 | bleed* OR hemorrhage OR haemorrhage OR "blood loss" OR (blood NEXT transfus*) OR hemoglobin* OR haemoglobin* OR hemosta* OR haemosta* | 150493 |
| #9 | #5 OR #6 OR #7 OR #8 | 155686 |
| #10 | MeSH descriptor: [Randomized Controlled Trial] explode all trees | 34 |
| #11 | (randomized OR randomised) NEXT trial OR ((randomized OR randomised) AND controlled AND trial) OR ((randomized OR randomised) NEXT controlled NEXT trial) OR "RCT" | 1140558 |
| #12 | #10 OR #11 | 1140558 |
| #13 | #4 AND #9 AND #12 with Publication Year from 2024 to 2025, in Trials | 3042 |
