# Supplementary material for: Definition, analysis, reporting, and interpretation of perioperative bleeding in randomized controlled trials: a methodological systematic review
Source: Perioper Med (Lond). 2026 Jul 2;15:80. doi: 10.1186/s13741-026-00715-z (PMC13595797; doi:10.1186/s13741-026-00715-z)
Supplement: Supplementary file 2 [file 13741_2026_715_MOESM3_ESM.docx]

**Additional File 3**

**Table S2. Timing of assessment by outcome definition used in the included studies (N = 116)**

| **Definitions** | **Timing of Assessment** |
| --- | --- |
| Suction volume only | During surgery [1–5] |
| Drain output | Up to hospital discharge [6–10] |
|  | Within 24 hrs after surgery[11] |
|  | 2 days after surgery [12] |
| Weighing of absorbent materials | During surgery [13,14] |
|  | Within 24-hrs after surgery[15] |
| Suction volume + Drain output | 2 days after surgery [16] |
| Suction volume + Absorbent material weight (objectively assessed) | During surgery [17–37] |
| Suction volume + Estimated blood absorbed by absorbent materials (subjectively assessed) | During surgery [38–42] |
| Suction volume + Absorbent material weight + Drain output | Up to hospital discharge [43] |
|  | Within 24 hrs after surgery [44,45] |
| Number of absorbent pads used | During surgery [46] |
| Hematocrit-based formula (Gross Formula) | Within 24 hrs after surgery [47–49] |
|  | 2 days after surgery [50] |
|  | 3 days after surgery [51–56] |
| Hemoglobin-based formula | Within 24 hrs after surgery[57] |
|  | 3 days after surgery [58] |
| Irrigation-based hemoglobin formula | During Surgery [59] |
| (Gross formula – DBL) + Allogenic blood transfusion | Up to hospital discharge [60] |
|  | 3 days after surgery [61] |
| Gross formula + Transfused blood | 2 days after surgery [62] |
|  | 3 days after surgery [63,64] |
| Total hemoglobin loss (%) | 2 days after surgery [65] |
|  | 3 days after surgery [66] |
|  | 4 days after surgery [67] |
| Wormald scale | During surgery [68] |
| Fromme–Boezaart scale | During surgery [69–73] |
| Modena bleeding score | During surgery [74] |
| Author-developed 3-Point visual field clarity scale | During surgery [75] |
| Hemostasis achieved | During surgery [76–80] |
| Time in minutes or seconds to achieve bleeding control | During surgery [81,82] |
| Author-defined 4-point scale assessing blood loss and transfusion need | During surgery [83] |
| Clinically detected bleeding during postoperative evaluation | Both up to and more than 30 days after surgery [84] |
| Visual severity or extent of ecchymosis or hematoma (rated by scale or measured area) | 7 days after surgery [85,86] |
|  | 10 days after surgery [87] |
| Transfusion rate (whether at least one transfusion was administered) | Up to hospital discharge [88–90] |
|  | Within 7 days after surgery [91,92] |
|  | Within 30 days after surgery [93,94] |
| Volume of blood transfused | Up to hospital discharge [95,96] |
| Number of units transfused | Within 24 hrs after surgery [97] |
| Composite of transfusion >2 RBC units or postoperative infection | Up to hospital discharge [98] |
| Difference between pre- and postoperative hemoglobin or hematocrit levels | Within 24 hrs after surgery [99–104] |
| Hemoglobin concentration in irrigation fluid | Within 24 hrs after surgery [105] |
| Rate of deviation from standard hemoglobin or hematocrits concentrations | During surgery [106] |
|  | Within 48 hrs after surgery [107] |
| Bleeding symptoms reported directly by the patient | 7 days after surgery [108] |
|  | 10-15 days after surgery [109] |
| Patient-reported bleeding duration (in days) | 14 days after surgery [110] |
| Incidence of hematoma (presence/absence in clinical examination) | Up to hospital discharge [111] |
|  | 30 days after surgery [112] |
| Need for additional treatment other than the main intervention | 14 days after surgery [110] |
| Need for re-intervention or hospitalisation | 28 days after surgery [113] |
| Universal Definition of Perioperative Bleeding (UDPB) | Within 24 hrs after surgery [114] |
| Clinically Significant Delayed Bleeding | 30 days after surgery [115] |

Note: References correspond to the included studies in which each definition was used.

**Figure S2. Proportion of different timings of perioperative bleeding assessment across outcomes (N = 116)**

**Figure S3. Distribution of Bleeding Outcome Definitions by Sample Size Quartiles (N = 116)**

**Figure S4. Distribution of Bleeding Outcome Definitions by Data Type (N = 116)**

**Figure S5. Distribution of Bleeding Outcome Definitions by Protocol-Specified Outcome Status (N = 116)**

**Figure S6. Distribution of Bleeding Outcome Definitions by Type of Intervention (N = 116)**

**Table S3. Sample Sizes and Analysis Practices by Outcome Type (N = 116)**

| **Characteristics** | **Categories** | **Total**  **N = 116** | **Continuous**  **(n = 77, 66.4%)** | **Binary**  **(n = 19, 16.4%)** | **Ordinal**  **(n = 13, 11.2%)** | **Count**  **(n = 3, 2.6%)** | **Composite**  **(n = 3, 2.6%)** | **Time-to-event**  **(n = 1, 0.9%)** |
| --- | --- | --- | --- | --- | --- | --- | --- | --- |
| **Size**  **Median [25^th^–75^th^ percentile]**  **(min–max)** | - | 105 [69.5–164.5]  (15–1384) | 100 [60–150]  (15–389) | 180 [128–353]  (69–1384) | 96 [70–130]  (50–310) | 50 [32–140]  (32–140) | 100 [78–234]  (78–234) | 160 |
| **Type of test** | Parametric | 69 (59.5%) | 54 (70.1%) | 8 (42.1%) | 4 (30.8%) | 2 (66.7%) | 0 (0.0%) | 1 (100.0%) |
|  | Non-parametric | 36 (31.0%) | 19 (24.7) | 5 (26.3%) | 8 (61.5%) | 1 (33.3%) | 3 (100.0%) | 0 (0.0%) |
|  | Both | 6 (5.2%) | 1 (1.3%) | 4 (21.05%) | 1 (7.7%) | 0 (0.0%) | 0 (0.0%) | 0 (0.0%) |
|  | N/A^a^ | 5 (4.3%) | 3 (3.9%) | 2 (10.5%) | 0 (0.0%) | 0 (0.0%) | 0 (0.0%) | 0 (0.0%) |
| **Adjustment for baseline covariates** | Yes | 18 (15.5%) | 8 (10.4%) | 8 (42.1%) | 1 (7.7%) | 0 (0.0%) | 0 (0.0%) | 1 (100.0%) |
|  | No | 98 (84.5%)_ | 69 (89.6%) | 11 (57.9%) | 12 (92.3%) | 3 (100.0%) | 3 (100.0%) | 0 (0.0%) |
| **Effect estimate reported** | Yes | 34 (29.3%) | 17 (22.1%) | 13 (68.4%) | 3 (23.1%) | 0 (0.0%) | 0 (0.0%) | 1 (100.0%) |
|  | No | 82 (70.7%) | 60 (77.9%) | 6 (31.6%) | 10 (76.9%) | 3 (100.0%) | 3 (100.0%) | 0 (0.0%) |
| **Confidence interval reported** | Yes | 28 (24.1%) | 11 (14.3%) | 13 (68.4%) | 3 (23.1%) | 0 (0.0%) | 0 (0.0%) | 1 (100.0%) |
|  | No | 88 (75.9%) | 66 (85.7%) | 6 (31.6%) | 10 (76.9%) | 3 (100.0%) | 3 (100.0%) | 0 (0.0%) |

^a^ Not reported, or no test was done, or Bayesian analysis.

Note: Percentages are column percentages.

**References**

1.	Akbari H, Nasiri Formi E, Alipour A, Delam H, Kavi E, Shahkarami N. A comparative study of the local effect of tranexamic acid and phenylephrine on the amount of bleeding in rhinoplasty: A randomized clinical trial. Caspian J Intern Med. 2024;15: 690–696. doi:10.22088/cjim.154.690

2.	Allahyani H, Bhat HS, Akhtar Z. Comparison of 5α-Reductase Inhibitors Pre-Treatment Versus TURP Alone in Transurethral Resection of the Prostate (TURP): A Comparative Study. Int J Life Sci Biotechnol Pharma Res. 2024;13: 235–237. doi:10.69605/ijlbpr_13.7.2024.43

3.	Park SJ, Lee JW, Hwang DW, Lee S, Yim GW, Song G, et al. Effect and Safety of Diluted Vasopressin Injection for Bleeding Control During Robot-assisted Laparoscopic Myomectomy in Reproductive Women With Uterine Fibroids: A Randomized Controlled Pilot Trial (VALENTINE Trial). In Vivo. 2024;38: 431–436. doi:10.21873/invivo.13456

4.	Sharifi M, Kiarudi MY, Gholamhoseinpoor-Omran S, Alipour M, Bakhtiari E. Intravenous Tranexamic Acid for Control of Bleeding during External Dacryocystorhinostomy under General Anesthesia: A Randomized Clinical Trial. JOVR. 2024;19: 340–346. doi:10.18502/jovr.v19i3.13947

5.	Singh AK, Khatri N. Efficacy of Tranexamic Acid in Controlling Bleeding Following Percutaneous Nephrolithotomy. International Journal of Toxicological and Pharmacological Research.

6.	Janowski C, Nguyen T, Da Silveira CB, Gulati S, Deka V, Thomas Gillespie, et al. A randomized controlled trial of the outcomes of Arista^TM^ hemostatic agent in robotic ventral hernia repair. J Robotic Surg. 2025;19: 81. doi:10.1007/s11701-025-02252-1

7.	Singh VA, Ong BK, Yasin NF. Oxidised cellulose in musculoskeletal oncology procedure: Does it reduce postoperative blood loss? Musculoskelet Surg. 2024;108: 483–489. doi:10.1007/s12306-024-00840-2

8.	Verma H, Jha CK, Singh PK, Sinha U, Ahmad S, Pandey JK, et al. Efficacy of perioperative systemic tranexamic acid along with topical hemocoagulase in decreasing axillary drain output in breast cancer patients undergoing axillary lymph node dissection: A randomized, double‐blind, placebo‐controlled, superiority trial. World j surg. 2024;48: 1433–1439. doi:10.1002/wjs.12189

9.	Zhou M, Li S, Zhang H, Lu Y. Does tranexamic acid reduce elbow swelling and improve early function following arthroscopic arthrolysis? A double-blind randomized controlled trial. Journal of Shoulder and Elbow Surgery. 2024;33: 2671–2679. doi:10.1016/j.jse.2024.06.009

10.	Fahmy M, Yasin E. Platelet rich plasma could replace tranexemic acid in total knee replacement? A comparative study between their postoperative- bleeding hemostatic effects. Current Orthopaedic Practice. 2024;35: 21–26. doi:10.1097/BCO.0000000000001238

11.	MikiÓ M, MilutinoviÓ D, AranÓeloviÓ B, StojakoviÓ N, ObradoviÓ M, PleÓaš-ĐuriÓ A, et al. Las ventajas del uso de ácido tranexámico en la reconstrucción del ligamento cruzado anterior: un ensayo controlado aleatorizado. CIRU. 2024;92: 13999. doi:10.24875/CIRU.23000578

12.	Hosseini A, Shahzamani M, Hashemabsdi A, Andalib A. The Effects of Albumin 20% and Hydroxyethyl Starch 6% on Bleeding and Interleukin-6 Levels as Priming Solutions for Cardiopulmonary Bypass: A Randomized Controlled Trial. J Tehran Heart Cent. 2024;19: 162–169.

13.	Chaichumporn T, Kanokkangsadal P, Sarovath A. Tranexamic Acid Subcutaneously Administered with Epinephrine and Lidocaine in Upper Blepharoplasty: A Randomized Double-Blind Control Trial. Aesth Plast Surg. 2024;48: 3076–3081. doi:10.1007/s00266-024-04112-z

14.	Tejada VFDS, Zhang L, Zogbi L. Efficacy and safety of topical application of tranexamic acid in patients undergoing reconstructive plastic surgery after excision of facial skin cancers: a randomised clinical trial. Rev Col Bras Cir. 2024;51: e20243761. doi:10.1590/0100-6991e-20243761-en

15.	Colclough RN, Almeland SK, Brekke RL, Spigset O, Nordgaard H, Pleym H, et al. Topical Tranexamic Acid on Donor Wounds in Burn Patients: A Randomized Placebo-controlled Trial. Plastic and Reconstructive Surgery - Global Open. 2024;12: e6074. doi:10.1097/GOX.0000000000006074

16.	Piette N, Beck F, Carella M, Hans G, Maesen D, Kurth W, et al. Oral as compared to intravenous tranexamic acid to limit peri-operative blood loss associated with primary total hip arthroplasty: A randomised noninferiority trial. European Journal of Anaesthesiology. 2024;41: 217–225. doi:10.1097/EJA.0000000000001950

17.	Abdelsalam WM, Hassan AA, Ghoniem BM, Elsharkawy MF, Shams GH. Dexmedetomidine Nasal Packing Versus Intravenous Dexmedetomidine for Controlling Bleeding During Turbinate Surgeries: A Randomized Double-Blind Trial. J Cell Mol Anesth. 2024;9. doi:10.5812/jcma-148840

18.	Ansari JR, Yarmosh A, Michel G, Lyell D, Hedlin H, Cornfield DN, et al. Intravenous Calcium to Decrease Blood Loss During Intrapartum Cesarean Delivery: A Randomized Controlled Trial. Obstetrics & Gynecology. 2024;143: 104–112. doi:10.1097/AOG.0000000000005441

19.	Arvind R, Poornima M, Mamatha S, Sowmya K, Chaitanya KT. Effect of Prophylactic Tranexamic Acid in Per-operative and Post-operative Blood Loss in Hysterectomy for Benign Condition: A Randomized Controlled Study. J Obstet Gynecol India. 2025;75: 43–51. doi:10.1007/s13224-024-01983-9

20.	Baghdasaryan MK, Mkhitaryan GK, Misakyan MS, Vermishyan VV, Tadevosyan AE, Bablumyan AYu, et al. Thulium laser versus cold steel tonsillectomy: a prospective pilot study in adult patients. Egypt J Otolaryngol. 2024;40: 26. doi:10.1186/s43163-024-00584-7

21.	Chen Q, Fu Y, Li Y, Cai H, Wang X, Wu Z, et al. Interim analysis of short-term outcomes after laparoscopic spleen-preserving distal pancreatectomy with or without preservation of splenic vessels: a randomised controlled trial. International Journal of Surgery. 2025;111: 617–627. doi:10.1097/JS9.0000000000001874

22.	Elmizadeh K, Beigom Khezri M, Pakniat H, Pandamouz V, Azh N, Molaverdikhani S. The Effect of Uterine Cooling During Cesarean Delivery on Blood Loss in Patients Undergoing Cesarean Section: A Randomized Controlled Trial. J Obstet Gynecol Cancer Res. 2024;9: 487–491. doi:10.30699/jogcr.9.5.487

23.	Heydari MB, Safdari M, Hemmatpoor B. Comparative Study of the Effects of Clonidine and Tranexamic Acid on Intraoperative Bleeding in Rhinoplasty: A Clinical Trial. JPRAS Open. 2024;41: 183–193. doi:10.1016/j.jpra.2024.04.013

24.	Huang C, Chen Z, Liu H, He J, Shen Y, Wu T, et al. The Effect of Thrombin-gelatin Matrix Compared With Absorbable Gelatin Sponge on Intraoperative Hemostasis in Unilateral Open-door Laminoplasty: A Randomized Controlled Trial. Spine. 2025;50: 285–293. doi:10.1097/BRS.0000000000005107

25.	Imaralu JO, Loto OM, Oguntade AF, Jagun OE, Ogunfunmilayo T, Akadri AA, et al. Adjunctive Uterine Incision Compression Versus Tourniquet Alone for Reduction of Blood Loss During Abdominal Myomectomy: A Randomized Controlled Trial. Ann Afr Surg. 2024;21: 23–34. doi:10.4314/aas.v21i1.4

26.	Lv H, Jiang X, Huang X, Wang W, Wu B, Yu S, et al. Nitroglycerin versus milrinone for low central venous pressure in patients undergoing laparoscopic hepatectomy: a double-blinded randomized controlled trial. BMC Anesthesiol. 2024;24: 244. doi:10.1186/s12871-024-02631-5

27.	Masoumzadeh M, Rahmani V, Sayyah-Melli M, Sani A. The effect of oxytocin, sublingual, and intrauterine misoprostol on blood loss in cesarean delivery: A randomized clinical trial. European Journal of Obstetrics & Gynecology and Reproductive Biology: X. 2025;25: 100369. doi:10.1016/j.eurox.2025.100369

28.	Meng C, Cao S, Li L, Xia L, Chu X, Jiang L, et al. Short-term outcomes of preoperative computed tomography angiography versus standard assessment in patients with BMI ≥ 25.0 kg/m2 undergoing laparoscopic gastrectomy: the GISSG20-01 randomized clinical trial. Gastric Cancer. 2025;28: 283–293. doi:10.1007/s10120-024-01580-9

29.	Mojaveraghili SB, Talebi F, Behnampour N. Comparison Effect of Clonidine and Magnesium Sulfate on Bleeding Volume during Septorhinoplasty: A Double-Blind, Randomized Clinical Trial. AACC. 2024 [cited 17 June 2025]. doi:10.18502/aacc.v10is1.17189

30.	Nasief H, Hassan A, AlDardeir N, Khadawardi K, Alwazzan AB, Alothmani H, et al. Oxytocin infusion is a safe uterine bleeding controller alternative to blood transfusion in patients undergoing abdominal myomectomy. Anaesth pain intensive care. 2024;28: 5–9. doi:10.35975/apic.v28i1.2298

31.	Naz E, Ali Khan FA, Zulfiqar B, Farrukh R, Naseem A, Isamail S. THE EFFECT OF SUBCUTANEOUS EPINEPHRINE DOSAGE ON BLOOD LOSS IN SURGICAL INCISION. J Ayub Med Coll Abbottabad. 2024;36: 64–67. doi:10.55519/JAMC-01-12671

32.	Srilekha N, Rao PSS. Effect of Systemic Tranexamic Acid on Intraoperative Bleeding During Tonsillectomy. 2024;18.

33.	Sumphaongern T, Chantarangsu S. Effect of Dexmedetomidine on Blood Loss and Patient Outcomes in Orthognathic Surgery: A Randomised Clinical Trial. International Dental Journal. 2025;75: 1961–1969. doi:10.1016/j.identj.2025.01.009

34.	Xu J, Zhao K, Xu S, Xu J, Sun B, Tong S, et al. Comparison of exsanguination and hemostasis devices for Limb surgery: a multicenter randomized controlled study. BMC Musculoskelet Disord. 2024;25: 679. doi:10.1186/s12891-024-07770-x

35.	Yl MK, Patil NS, Mohapatra N, Sindwani G, Dhingra U, Yadav A, et al. Temporary Portocaval Shunt Provides Superior Intraoperative Hemodynamics and Reduces Blood Loss and Duration of Surgery in Live Donor Liver Transplantation: A Randomized Control Trial. Annals of Surgery. 2024;279: 932–944. doi:10.1097/SLA.0000000000006200

36.	Zaman B, Mohseni M, Noorizad S, Jalali Motlagh S, Amiraslani T, Sayyahi M. Evaluating the Effect of Pressure-Controlled Versus Volume-Controlled Ventilation Modes on Intraoperative Bleeding in Rhinoplasty: A Randomized Clinical Trial. Anesth Pain Med. 2024;14. doi:10.5812/aapm-151582

37.	Sinha N, Rajbhar S, Thakur P, Agrawal S, Singh V. Role of prophylactic tranexamic acid in reducing blood loss during cesarean section: A double-blind placebo-controlled randomized controlled trial. Journal of Family Medicine and Primary Care. 2024;13: 1760–1765. doi:10.4103/jfmpc.jfmpc_1541_23

38.	Adibparsa M, Ghazavi M, Shetabi H. Impact of Topical Tranexamic Acid on Bleeding Control in Rhinoplasty. WJPS. 2024;13: 66–74. doi:10.61186/wjps.13.3.66

39.	Calim M, Karaaslan K, Yilmaz S, Senturk E, Deniz H, Akbas S. The Effects of Hypocapnia and Hypercapnia on Intraoperative Bleeding, Surgical Field Quality, and Surgeon Satisfaction Level in Septorhinoplasty: A Prospective Randomized Clinical Study. Aesth Plast Surg. 2024;48: 167–176. doi:10.1007/s00266-023-03433-9

40.	Kamali A, Naseri N, Zamani F, Anosheh N, Rakei S. The effect of dexmedetomidine, remifentanil and metoral in reducing patient bleeding during rhinoplasty surgery. ITJ. 2024;27. doi:10.5935/0946-5448.20230024

41.	Kargar-soleimanabad S, Khormali A, Godazandeh F, Najafi S. Effect of oral tranexamic acid on postoperative bleeding in spinal surgery: a randomized controlled trial. Annals of Medicine & Surgery. 2024;86: 4483–4487. doi:10.1097/MS9.0000000000001820

42.	Vaezi M, Zarei R, Azizi H. Comparison of Maylard and Cherney incisions’ outcomes in hysterectomy surgery for benign indications: a double-blind randomized controlled trial. Eur J Med Res. 2025;30: 56. doi:10.1186/s40001-025-02311-1

43.	Olaleye AA, Adebayo JA, Eze JN, Ajah LO, Anikwe CC, Egede JO, et al. Efficacy of Tranexamic Acid in Reducing Myomectomy-Associated Blood Loss among Patients with Uterine Myomas at Federal Teaching Hospital Abakaliki: A Randomized Control Trial. Gaspar R, editor. International Journal of Reproductive Medicine. 2024;2024: 1–8. doi:10.1155/2024/2794052

44.	Abdelsami Aiad A, Gaballah KM, Sultan WA. Perioperative oral pregabalin or clonidine in patients undergoing lumbar spine posterior fusion: A randomized controlled trial. Egyptian Journal of Anaesthesia. 2024;40: 127–134. doi:10.1080/11101849.2024.2319523

45.	Elbaseet HM, Aldeen AJ, Irahim AKH. Efficacy of intraoperative use of tranexamic acid in reducing blood loss from telescoping nail application in osteogenesis imperfecta. A randomized controlled trial. Orthopaedics & Traumatology: Surgery & Research. 2025;111: 103927. doi:10.1016/j.otsr.2024.103927

46.	Kovalis N, Graffi S, Safuri S, Shapira Y, Ben-David G, Mimouni M. Duration of Bare Sclera Pterygium Surgery Combined with Mitomycin C with and Without Tranexamic Acid: A Randomized Double-Blind Controlled Trial. Journal of Ocular Pharmacology and Therapeutics. 2024;40: 675–679. doi:10.1089/jop.2024.0068

47.	Atef A, Shehata HSEAM, Bassiouny YA, Al-Inany HG. Comparative study between the roles of intrauterine misoprostol versus the sublingual route for prevention of postpartum blood loss in elective cesarean sections: a randomized controlled trial. BMC Pregnancy Childbirth. 2024;24: 710. doi:10.1186/s12884-024-06889-y

48.	Güngördük K, Ünver HC, Uyar BŞ, Ergü BN, Gülseren V, Özdemir İA. Efficacy of chitosan powder in reducing vaginal bleeding following a loop electrosurgical excision procedure: A randomized control trial. Intl J Gynecology & Obste. 2025;169: 240–246. doi:10.1002/ijgo.16055

49.	Krauss ES, Segal A, Simonson BG, Dengler N, Cronin M. Comparison of the Unipolar Electrocautery and the Bipolar Sealer in Reducing Blood Loss in Total Knee Arthroplasty: A Prospective, Randomized, Noninferiority Study. Arthroplasty Today. 2024;29: 101509. doi:10.1016/j.artd.2024.101509

50.	Neumann BG, Metgud MC, Hoffman MK, Patil K, Savanur M, Hanji V, et al. Tranexamic acid to reduce blood loss in women at high risk of postpartum hemorrhage undergoing cesarean delivery—a randomized controlled trial. AJOG Global Reports. 2024;4: 100316. doi:10.1016/j.xagr.2024.100316

51.	Alasaad H, Ibrahim J. Evaluation of efficacy and safety of perioperative tranexamic acid during Primary Total Knee Arthroplasty: A randomized, Clinical trial. Orthopedic Reviews. 2024;16. doi:10.52965/001c.118441

52.	Han G, Cai L, Wang Q, Li Q, Kang P. A randomized controlled trial comparing carbazochrome sodium sulfonate and tranexamic acid in reducing blood loss and inflammatory response after simultaneous bilateral total hip arthroplasty. J Orthop Surg (Hong Kong). 2024;32: 10225536241293544. doi:10.1177/10225536241293544

53.	Li B, Pan W, Ma J, Huang Y. Hemostatic effect of oxidized regenerated cellulose vs. topical tranexamic acid in total knee arthroplasty—a prospective randomized controlled trial. Front Surg. 2025;11: 1515610. doi:10.3389/fsurg.2024.1515610

54.	Lou Z, Jiang K, Xia S, Chen S, Jiang Y, Zhu J, et al. Efficacy and Safety of Tranexamic Acid on Hidden Blood Loss in Osteoporotic Vertebral Compression Fractures Patients Treated with Percutaneous Kyphoplasty: A Prospective Randomized Controlled Trial. TCRM. 2024;Volume 20: 907–917. doi:10.2147/TCRM.S494728

55.	Ye S, Gan Y, Li Q, Cai L, Kang P. Efficacy and Safety of Bone Wax Application at Different Time Points to Reduce Postoperative Blood Loss in Total Knee Arthroplasty: A Prospective Randomized Controlled Trial. Orthopaedic Surgery. 2024;16: 2447–2453. doi:10.1111/os.14177

56.	Ye S, Luo Y, Li Q, Cai L, Kang P. Efficacy of Different Doses of Intra‐Articular Tranexamic Acid for Reducing Blood Loss and Lower Limb Swelling After Total Knee Arthroplasty: A Prospective, Randomized, Controlled Trial. Orthopaedic Surgery. 2025;17: 733–743. doi:10.1111/os.14317

57.	Hosseini-Monfared P, Mirahmadi A, Sarzaeem MM, Pourshahryari S, Aminnia P, Poursalehian M, et al. Ascorbic Acid Reduces the Blood Boss After Total Knee Arthroplasty: Insights From a Randomized Controlled Trial. Arthroplasty Today. 2025;32: 101618. doi:10.1016/j.artd.2025.101618

58.	Bosilah AH, Hussein M, Alboghdady MA, Zaky A, Shafik Almorsy A, Taha WS, et al. To B or not to B: the application of uterine compression sutures to reduce blood loss after myomectomy. pm. 2024;23: 21–24. doi:10.5114/pm.2024.136954

59.	Rohith G, Das MK, Rao PB, Gaur AS, Mandal S, Ramasubbu MK, et al. Efficacy of Low Dose Intravenous Epinephrine Infusion in Improving Perioperative Outcomes in Patients Undergoing Transurethral Resection of Prostate: A Prospective Parallel Arm Double-Blind Randomized Control Trial. Urology. 2024;184: 169–175. doi:10.1016/j.urology.2023.11.023

60.	Dong W, Tang Y, Lei M, Ma Z, Zhang X, Shen J, et al. The effect of perioperative sequential application of multiple doses of tranexamic acid on postoperative blood loss after PLIF: a prospective randomized controlled trial. International Journal of Surgery. 2024;110: 2122–2133. doi:10.1097/JS9.0000000000001083

61.	Luo G, Liu J, Ni W, Huang W. The role of post-traumatic antifibrinolysis in the perioperative blood management of elderly patients with intertrochanteric fractures treated with PFNA: A randomised controlled trial. Injury. 2024;55: 111877. doi:10.1016/j.injury.2024.111877

62.	Naderi MA, Moghadam AD, Mobayen M, Rimaz S, Haghani-Dogahe Z, Roudsarabi S, et al. Effects of intravenous tranexamic acid on bleeding during burn surgery: A double-blinded randomized clinical trial. Burns. 2025;51: 107270. doi:10.1016/j.burns.2024.09.009

63.	Luo X-P, Peng J, Zhou L, Liao H, Jiang X-C, Tang X, et al. Intramedullary administration of tranexamic acid reduces bleeding in proximal femoral nail antirotation surgery for intertrochanteric fractures in elderly individuals: A randomized controlled trial. Chinese Journal of Traumatology. 2025;28: 201–207. doi:10.1016/j.cjtee.2024.01.006

64.	Xie J, Huang Q, Huang Z, Wang F, Liu J, Tian H, et al. High dose of tranexamic acid infusion in primary total knee arthroplasty: A randomized multicenter clinical trail. Chinese Medical Journal. 2024;137: 359–361. doi:10.1097/CM9.0000000000002952

65.	Pinsornsak P, Thaveekitikul R, Pinsornsak P, Tanariyakul Y. Comparative effectiveness of combined peri-articular and intra-articular injection versus intravenous and intra-articular injection of tranexamic acid in total knee arthroplasty: a randomized controlled trial study. Arch Orthop Trauma Surg. 2024;144: 2753–2759. doi:10.1007/s00402-024-05337-y

66.	Chaiyakit P, Kabkaew P, Hongku N, Wattanapreechanon P. Comparison of total blood loss between limited tourniquet use and conventional tourniquet use in total knee arthroplasty: a randomized controlled trial. BMC Musculoskelet Disord. 2024;25: 918. doi:10.1186/s12891-024-08058-w

67.	Miralles-Muñoz FA, Martin-Grandes R, Pineda-Salazar M, Bello-Tejeda LL, De La Pinta-Zazo C, Farrer-Muñoz P. Preoperative dose of intravenous tranexamic acid safely reduces blood loss and transfusion in patients undergoing hip hemiarthroplasty for femoral neck fracture. A randomized controlled trial. Acta Orthop Belg. 2024;90: 403–408. doi:10.52628/90.3.11635

68.	Abdelaziz T, Elsayed H, Salem M, Mahmoud NY. ”The efficacy of oral versus intravenous tranexamic acid in functional endoscopic sinus surgery”. A prospective, randomized, controlled trial. Egyptian Journal of Anaesthesia. 2024;40: 105–111. doi:10.1080/11101849.2024.2313414

69.	Ghazaly HF, Khalefa MM, Sedeek MA, Hasanen MK. Sphenopalatine ganglion block with lidocaine versus bupivacaine for optimizing intraoperative bleeding and surgical field visibility during endoscopic septoplasty: a randomized double-blind controlled trial. Anaesth pain intensive care. 2024;28: 92–99. doi:10.35975/apic.v28i1.2393

70.	Mohamed Hassan A, Nashaat Mohammed M, Abdo Mousa S, Elsharkawy RA. A comparative study between the effect of intravenous versus intranasal dripping of dexmedetomidine on intraoperative blood loss during functional endoscopic nasal sinus surgery. Egyptian Journal of Anaesthesia. 2024;40: 171–178. doi:10.1080/11101849.2024.2331359

71.	Singh R, Choudhary A, Singh S, Kumar H. A comparative study of two different doses of dexmedetomedine as an adjuvant to lignocaine in infiltration block for tympanoplasty: a triple-blinded, prospective, randomized controlled trial. Anesth Pain Med. 2024;19: 310–319. doi:10.17085/apm.24105

72.	Vaghardoost R, Ahmadi Dahaj A, Haji Mohammad M, Ghadimi T, Forghani SF, Naderi Gharahgheshlagh S. Evaluating the Effect of Tranexamic Acid Local Injection on the Intraoperative Bleeding Amount and the Postoperative Edema and Ecchymosis in Primary Rhinoplasty Patients: A Randomized Clinical Trial. Aesth Plast Surg. 2024;48: 702–708. doi:10.1007/s00266-023-03441-9

73.	Zhang K, Wang L, Qi F, Meng T. Hypotensive Levels on Endoscopic Sinus Surgery Visibility: A Randomized Non‐Inferiority Trial. The Laryngoscope. 2024;134: 569–576. doi:10.1002/lary.30867

74.	Zhang Y, Han L, Ding W, Gao L, Feng Y, An H. Intravenous tranexamic acid significantly improved visualization and shortened the operation time in microscopic middle ear surgery: a randomized controlled trial. International Journal of Surgery. 2024;110: 4170–4175. doi:10.1097/JS9.0000000000001366

75.	Kunze KN, Madjarova S, Olsen R, Smolarsky R, Menta S, Baldwin R, et al. Intravenous Tranexamic Acid Does Not Improve Visual Field Clarity During Hip Arthroscopy: A Double-Blind, Randomized, Placebo-Controlled Trial. Arthroscopy: The Journal of Arthroscopic & Related Surgery. 2025; S074980632500012X. doi:10.1016/j.arthro.2025.01.008

76.	An S, Kwon W-K, Choi I, Lee J-B, Kim J, Hur JW. Evaluating the Efficacy and Safety of Hemofence (Thorombin Cross-Linked Sodium Hyaluronate Gel Matrix) in Hemostasis for Intractable Exudative Bleeding in Spinal Surgery: A Multicenter, Randomized, Phase III Clinical Trial. Neurospine. 2024;21: 1004–1013. doi:10.14245/ns.2448024.012

77.	Balanescu L, Gajdobranski D, Sretenović A, Kalinova K, Vajda P, Hanna K, et al. A Phase 3, Randomized, Active-controlled, Single-blind Clinical Trial to Evaluate the Efficacy of Fibrin Sealant Grifols in Achieving Hemostasis in Pediatric Surgery. Journal of Pediatric Surgery. 2024;59: 161639. doi:10.1016/j.jpedsurg.2024.07.024

78.	House MG, Kim R, Tseng EE, Kaufman RP, Moon MR, Yopp A, et al. Evaluating the safety and efficacy of a novel polysaccharide hemostatic system during surgery: A multicenter multispecialty prospective randomized controlled trial. Surgery Open Science. 2024;19: 205–211. doi:10.1016/j.sopen.2024.04.009

79.	Luo P, Lai Q, Xu L, Chen Z, Guo R, Xie B, et al. Efficacy and safety evaluation of a novel hemostatic gelatin matrix for intraoperative hemostasis: a prospective, randomized controlled trial. Sci Rep. 2024;14: 27324. doi:10.1038/s41598-024-77487-3

80.	Shady NW, Taha AA, Sallam HF. The adjunctive role of temporary uterine packing combined with topical tranexamic acid for reducing blood loss during haemorrhagic caesarean delivery: a randomized controlled trial. Ital J Gynaecol Obstet. 2024;36: 370. doi:10.36129/jog.2024.151

81.	Kang O-J, Nam J-H, Park J-Y. Comparative Analysis of Electrosurgical Energy and Hemostatic Sealant for Hemostasis in Laparoscopic Ovarian Cystectomy: A Randomized Controlled Phase III Study. Journal of Minimally Invasive Gynecology. 2025;32: 177–184. doi:10.1016/j.jmig.2024.10.001

82.	Kenny S, Gabra H, Hall NJ, Flageole H, Illie B, Barnett E, et al. A Study of Safety and Effectiveness of Evicel Fibrin Sealant as an Adjunctive Hemostat in Pediatric Surgery. Eur J Pediatr Surg. 2024;34: 512–521. doi:10.1055/s-0044-1785443

83.	Sarode R, Goldstein JN, Simonian G, Hinterberger D, Matveev D, Gareis M, et al. Vitamin K Antagonist Reversal for Urgent Surgery Using 4-Factor Prothrombin Complex Concentrates: A Randomized Clinical Trial. JAMA Netw Open. 2024;7: e2424758. doi:10.1001/jamanetworkopen.2024.24758

84.	Su T, Lai D, Wu Y, Gu C, He S, Meng C, et al. COMPARISON OF THE EFFICACY AND SAFETY OF RANIBIZUMAB 0.5 MG VERSUS 1.0 MG WITH PARS PLANA VITRECTOMY FOR THE TREATMENT OF PROLIFERATIVE DIABETIC RETINOPATHY: A Randomized Controlled Trial. Retina. 2024;44: 680–688. doi:10.1097/IAE.0000000000003998

85.	Marous CL, Farhat OJ, Cefalu M, Rothschild MI, Alapati S, Wladis EJ. Effects of Preoperative Intravenous Versus Subcutaneous Tranexamic Acid on Postoperative Periorbital Ecchymosis and Edema Following Upper Eyelid Blepharoplasty: A Prospective, Randomized, Double-Blinded, Placebo-Controlled, Comparative Study. Ophthalmic Plastic & Reconstructive Surgery. 2024;40: 523–532. doi:10.1097/IOP.0000000000002633

86.	Paramo R, Cheng T, Malik A, Fan J, Barmettler A. Effect of Tranexamic Acid on Intra- and Postoperative Bleeding in Eyelid Surgery: A Prospective, Randomized, Multicenter, Double-Masked, Control Trial. Ophthalmic Plastic & Reconstructive Surgery. 2024;40: 331–335. doi:10.1097/IOP.0000000000002583

87.	Cano Valls A, Niebla M, Diago C, Domingo R, Tolosana JM, Pérez S. Efficacy of Hypothermic Compression Bandages in Cardiac Device Surgical Wounds: A Randomized Controlled Trial. Adv Skin Wound Care. 2024;37: 1–7. doi:10.1097/ASW.0000000000000201

88.	Gao S, Liu G, Wang J, Zhang Q, Wang J, Teng Y, et al. A modified low-priming cardiopulmonary bypass system in patients undergoing cardiac surgery with medium risk of transfusion: A randomized controlled trial. Heliyon. 2024;10: e31388. doi:10.1016/j.heliyon.2024.e31388

89.	Mastroianni R, Tuderti G, Ferriero M, Anceschi U, Bove AM, Brassetti A, et al. Robot-assisted Radical Cystectomy with Totally Intracorporeal Urinary Diversion Versus Open Radical Cystectomy: 3-Year Outcomes from a Randomised Controlled Trial. European Urology. 2024;85: 422–430. doi:10.1016/j.eururo.2024.01.018

90.	Saour M, Blin C, Zeroual N, Mourad M, Amico M, Gaudard P, et al. Impact of a bundle of care (intravenous iron, erythropoietin and transfusion metabolic adjustment) on post-operative transfusion incidence in cardiac surgery: a single-centre, randomised, open-label, parallel-group controlled pilot trial. The Lancet Regional Health - Europe. 2024;43: 100966. doi:10.1016/j.lanepe.2024.100966

91.	Karanicolas PJ, Lin Y, McCluskey SA, Tarshis J, Thorpe KE, Wei A, et al. Tranexamic Acid in Patients Undergoing Liver Resection: The HeLiX Randomized Clinical Trial. JAMA. 2024;332: 1080. doi:10.1001/jama.2024.11783

92.	Khatib Y, Bal G, Liu R, Ashaia W, Sorial R. A randomised controlled trial assessing the effect of tranexamic acid on post-operative blood transfusions in patient with intra-capsular hip fractures treated with hemi- or total hip arthroplasty. Arch Orthop Trauma Surg. 2024;144: 3095–3102. doi:10.1007/s00402-024-05325-2

93.	Breau RH, Lavallée LT, Cagiannos I, Momoli F, Bryson GL, Kanji S, et al. Tranexamic Acid During Radical Cystectomy: A Randomized Clinical Trial. JAMA Surg. 2024;159: 1355. doi:10.1001/jamasurg.2024.4183

94.	Martel G, Carrier FM, Wherrett C, Lenet T, Mallette K, Brousseau K, et al. Hypovolaemic phlebotomy in patients undergoing hepatic resection at higher risk of blood loss (PRICE-2): a randomised controlled trial. The Lancet Gastroenterology & Hepatology. 2025;10: 114–124. doi:10.1016/S2468-1253(24)00307-8

95.	Ismail TI, Mahrous RS. Prophylactic cryoprecipitate transfusion in patients undergoing scoliosis surgery: A randomised-controlled trial. Journal of Perioperative Practice. 2024;34: 60–69. doi:10.1177/17504589221132393

96.	Ravichandran AK, Kalirajan R, Nambiraj G, Kanthan SVL, Raju M, Tiwary T, et al. Effectiveness of Tranexamic Acid to Prevent Blood Loss in Patients Undergoing Hip Arthroplasty: A Randomised Controlled Study. JCDR. 2025 [cited 17 June 2025]. doi:10.7860/JCDR/2025/76185.20675

97.	Mahdy AM, Al-Taher EM, Helmy AM, Kamhawy GA, Tawadros PZ, Fayad EA, et al. Rotational Thromboelastometry Guided Transfusion Protocol Versus Classic Transfusion Protocol in Reducing Blood Components Transfusion in Adult Patients Undergoing Elective Cardiac Surgery: A Randomized Clinical Trial. Sri Lankan J Anaesthesiol. 2025;33: 36–45. doi:10.4038/slja.v33i01.9386

98.	Kiviniemi TO, Anttila V, Pälve K, Vesanen M, Lehto J, Malmberg M, et al. Ferric carboxymaltose in reducing blood transfusions and infections after cardiac surgery. JTCVS Open. 2024;22: 332–344. doi:10.1016/j.xjon.2024.09.009

99.	Kundargi VS, Patil S, Patil SB, Vaidya MK, Patil BS, Shariff AN, et al. Role of tranexamic acid via intravenous and irrigation fluid route in controlling TURP-associated bleeding. Afr J Urol. 2024;30: 68. doi:10.1186/s12301-024-00473-8

100.	Langar E, Haddad F, Ben Hadj Youssef A, Kammoun E, Saddem I, Mebazaa MS. Prevention of bleeding in total hip and knee replacement: Contribution of combined route in tranexamic acid administration. Tunis Med. 2025;103: 225–232. doi:10.62438/tunismed.v103i2.4991

101.	Lee AYM, Neo SH, Brodie BA, Ng TK, Aslim EJ, Chin ZY, et al. Preoperative prostatic artery embolization before transurethral resection of the prostate for prostate glands larger than 80 mL: the first randomized controlled trial. Prostate International. 2025;13: 22–27. doi:10.1016/j.prnil.2024.10.004

102.	McDermott CD, Lovatsis D, Wang S, Huszti E, Wadsworth K. Does Intravenous Tranexamic Acid Reduce Blood Loss at the Time of Total Colpocleisis? A Randomized Double-Blind Placebo-Controlled Trial. Journal of Minimally Invasive Gynecology. 2025;32: 555–561. doi:10.1016/j.jmig.2025.01.005

103.	Nouralizadeh A, Darzi MM, Adib A, Kashi AH. An assessment of the efficacy and safety of balloon nephrostomy traction in minimizing postoperative bleeding of percutaneous nephrolithotomy: a randomized controlled clinical trial. Langenbecks Arch Surg. 2025;410: 26. doi:10.1007/s00423-024-03599-z

104.	Omar M, Ibrahim TAA, Sultan S, El-Gharabawy M, Noureldin Y, Hamri SB, et al. Evaluating the safety of bipolar nephrostomy tract cauterization “BNTC” towards a safe tubeless percutaneous nephrolithotomy: a randomized controlled trial. Urolithiasis. 2024;52: 106. doi:10.1007/s00240-024-01575-2

105.	Diab T, El Hamshary SA, Abou Elezz A, El-Dakhakhny AS. Intraprostatic Injection of Tranexamic Acid Decrease Blood Loss During Monopolar Transurethral Resection of the Prostate: A Randomized Controlled Clinical Trial. Urology. 2024;191: 119–126. doi:10.1016/j.urology.2024.05.015

106.	Park S-K, Hur C, Kim Y-W, Yoo S, Lim Y-J, Kim J-T. Noninvasive hemoglobin monitoring for maintaining hemoglobin concentration within the target range during major noncardiac surgery: A randomized controlled trial. Journal of Clinical Anesthesia. 2024;93: 111326. doi:10.1016/j.jclinane.2023.111326

107.	Demirpolat MT, Islam MM, Bacaksiz ME, Ertekin SC, Sisik A. Comparison of Early Postoperative Outcomes of Omentopexy and Clips along the Staple Line During Laparoscopic Sleeve Gastrectomy: A Randomized Study. OBES SURG. 2024;34: 4116–4124. doi:10.1007/s11695-024-07543-4

108.	Ali G, Ali A, Singh AK. Intraoperative Dexamethasone Single Dose and Risk of Postoperative Haemorrhage in Tonsillectomy Patients. International Journal of Pharmaceutical and Clinical Research.

109.	Radtke S, Arms R, Son MA, Sanchez S, Singh V, Bencomo M, et al. Reducing patient’s perception of postoperative vaginal bleeding after laparoscopic hysterectomy via independent closure of the vaginal cuff angles (RCT). European Journal of Obstetrics & Gynecology and Reproductive Biology. 2024;294: 111–116. doi:10.1016/j.ejogrb.2024.01.019

110.	Kim J, Park J-Y. Hemostatic Efficacy of TachoSil in Loop Electrosurgical Excisional Procedure: A Prospective Randomized Controlled Study. J Low Genit Tract Dis. 2025;29: 123–130. doi:10.1097/LGT.0000000000000867

111.	Yeow M, Tan L, Fatt SLK, Mahipal M, Parameswaran R, Loo L, et al. Cessation vs. no cessation of acetylsalicylic acid preoperatively in laparoscopic totally extraperitoneal inguinal hernia repair (CAPTAIN): interim report from a multicentre, single-blind, randomised controlled trial. Hernia. 2025;29: 78. doi:10.1007/s10029-025-03263-5

112.	Yao A, Wang F, Benacquista T, Draper LB, Garfein ES, Monaco C, et al. Topical Tranexamic Acid Does Not Reduce Hematoma in Reduction Mammaplasty: A Double-Blind Randomized Controlled Trial. Plastic & Reconstructive Surgery. 2024;154: 30–37. doi:10.1097/PRS.0000000000010952

113.	Chiang H-C, Chen P-J, Yang E-H, Kuo T-L, Hsieh M-T, Kang J-W, et al. Clinical Trial: Precise Administration of Sucralfate Powder in Prevention of Delayed Postpolypectomy Bleeding. A Randomized Controlled Trial. Clin Transl Gastroenterol. 2025;16: e00818. doi:10.14309/ctg.0000000000000818

114.	Bratt S, Dimberg A, Kastengren M, Lilford RD, Svenarud P, Sartipy U, et al. Bleeding in minimally invasive versus conventional aortic valve replacement. J Cardiothorac Surg. 2024;19: 349. doi:10.1186/s13019-024-02667-1

115.	Drews J, Zachäus M, Kleemann T, Schirra J, Cahyadi O, Möschler O, et al. Multicentre randomised controlled trial of a self-assembling haemostatic gel to prevent delayed bleeding following endoscopic mucosal resection (PURPLE Trial). Gut. 2025; gutjnl-2024-334229. doi:10.1136/gutjnl-2024-334229
